# Unravelling Genes and Pathways Implicated in Working Memory of Schizophrenia in Han Chinese

**DOI:** 10.3390/ijms16012145

**Published:** 2015-01-20

**Authors:** Hongyan Ren, Chengcheng Zhang, Chaohua Huang, Na Li, Mingli Li, Yinfei Li, Wei Deng, Xiaohong Ma, Bo Xiang, Qiang Wang, Tao Li

**Affiliations:** 1 Mental Health Center, West China Hospital, Sichuan University, 28 Dian Xin Nan Road, Chengdu 610041, China; E-Mails: sandyren_6@hotmail.com (H.R.); zhangcc89@foxmail.com (C.Z.); chaohua118@foxmail.com (C.H.); linaxdrs@163.com (N.L.); xinzhishui@tom.com (M.L.); li-yinfei@163.com (Y.L.); mrdengwei@163.com (W.D.); maxiaohong@scu.edu.cn (X.M.); 2 State Key Laboratory of Biotherapy, Psychiatric Laboratory, West China Hospital, Sichuan University, 1 Ke Yuan 4 Road, Hi-Tech Developmental Zone, Chengdu 610041, China; E-Mail: xiangboyulan2012@gmail.com

**Keywords:** working memory, delayed-matching-to-sample test, schizophrenia, genome-wide association study, pathway

## Abstract

Working memory deficit is the core neurocognitive disorder in schizophrenia patients. To identify the factors underlying working memory deficit in schizophrenia patients and to explore the implication of possible genes in the working memory using genome-wide association study (GWAS) of schizophrenia, computerized delay-matching-to-sample (DMS) and whole genome genotyping data were obtained from 100 first-episode, treatment-naïve patients with schizophrenia and 140 healthy controls from the Mental Health Centre of the West China Hospital, Sichuan University. A composite score, delay-matching-to-sample total correct numbers (DMS-TC), was found to be significantly different between the patients and control. On associating quantitative DMS-TC with interactive variables of groups × genotype, one SNP (rs1411832), located downstream of YWHAZP5 in chromosome 10, was found to be associated with the working memory deficit in schizophrenia patients with lowest *p*-value (*p* = 2.02 × 10^−7^). ConsensusPathDB identified that genes with SNPs for which *p* values below the threshold of 5 × 10^−5^ were significantly enriched in GO:0007155 (cell adhesion, *p* < 0.001). This study indicates that working memory, as an endophenotype of schizophrenia, could improve the efficacy of GWAS in schizophrenia. However, further study is required to replicate the results from our study.

## 1. Introduction

Schizophrenia is a psychiatric disorder that affects about 1% of the world population [1]. Both win and adoption studies have shown that genetic factors play an important role in the pathogenesis of schizophrenia (heritability close to 0.8) [2]. Schizophrenia, having a high heritability, is a multi-dimensional disorder that cannot be explained by Mendelian genetics. It is now generally agreed that it is caused by combined effects of hundreds or thousands of gene variants with modest effects [3]. Advances in whole-genome genotyping and related analysis methods have enabled identification of these variants. Since 2009, many genome-wide association studies (GWAS), with case-control designs, have been carried out to study the genetic architecture underlying schizophrenia [4,5,6]. The results from a GWA study with the largest sample size so far, identified 108 variants that are associated with schizophrenia. Although some variants, such as ones in ZNF804A and variants in major histocompatibity complex (MHC), have been reported to be replicated, many SNPs were found to be non-overlapping in this study [7]. The inconsistency was attributed to factors such as population stratification, sample size, and clinical heterogeneity, with clinical heterogeneity being the most difficult confounding factor in case-control GWASs. Unlike other complex disorders, diagnosis of schizophrenia lacks reliable biomarkers and animal models. In fact, most of its diagnoses are based on subjective judgments from clinical practitioners. Additionally, lack of objective laboratory measures tends to create a barrier between the disorder diagnosis and its management. Although large sample size is a good strategy to map related variants, many case-control studies still failed to produce satisfactory results. Alternatively, various researchers used quantitative traits (QTs) as endophenotype or intermediate phenotype in order to enhance the efficacy of the GWAS in schizophrenia [8,9]. In comparison to dichotomous phenotype (subjective and random-prone diagnoses), QT is easy to measure and can be standardized. In a continuum from gene to clinical outcome, quantitative pathological changes are supposed to be more proximal for genetic underpinnings of the disease. Various studies have shown that QTs improve the efficacy of GWAS [6,9,10,11,12]. Further, they were also found to be beneficial in mapping the genes associated with schizophrenia. Dickinson *et al.* [13] used g score, a composite score combining six neuro-cognitive dimensions as QTs in a genome association study to map potential genetic variants associated with schizophrenia. This approach led to unveiling the effects of SCN2A on general cognitive ability, brain physiology, and mRNA expression in schizophrenia. Potkin *et al.* [14] carried out a genome-wide study on schizophrenia by using QTs with results showing that variants in six genes (POU3F2, TRAF, GPC1, POU3F2, TRAF, and GPC1) were associated significantly with working memory task-related bold signal of functional MRI in schizophrenia, with a *p*-value threshold of 10^−6^. Furthermore, they identified these six genes/regions involved in pathways related to neurodevelopment and response to stress. These studies demonstrated the efficacy of using QTs as endophenotype in exploring the pathogenesis of schizophrenia.

Previous studies have demonstrated that working memory deficit exists both in schizophrenic patients and their biological relatives [15,16,17]. In the present study, we used the total correct numbers of delay-matching-to-sample (DMS-TC) as QTs, which is a composite score generated from delay-matching-to-sample (DMS) test in the Cambridge Neuropsychological Test Automated Battery (CANTAB), to explore common genetic variants underlying the working memory deficit in schizophrenia by using a hypothesis-free GWAS analysis. Furthermore, we used ConsensusPathDB (available on line: http://consensuspathdb.org/), one of the most widely applied pathway databases, to analyze pathway over-representation of genes that the associated variants are located in or close to. It is assumed that this downstream strategy will increase validity of the study and might shed light on the mechanism of how these biological pathways bridge genes with neurocognitive deficits in schizophrenia.

## 2. Results

### 2.1. Demographic Characteristics and Delayed-Matching-to-Sample (DMS) Test

A hundred and forty first-episode and drug-naive patients with schizophrenia and 100 healthy controls were included in the study. There was no difference between patients and controls in term of sex, age, and education years (Table 1). Nineteen scores of 125 individuals were successfully uploaded into the result dataset (67 controls, 58 cases) after DMS test. Of these 19 measurement scores from DMS, 10 scores remained significantly different between patients and healthy controls with age, sex, and education year being adjusted for. However, after multiple testing, only one of these scores, DMS-TC, survived Bonferroni correction, as shown in Table 1 (*p* < 0.05). From the study, it was observed that DMS-TC score can be used for QTs in subsequent analysis.

**Table 1:** Summary of Demographic characteristics and Delayed-matching-to-sample (DMS) results.

Demographic Characteristics and DMS Measures	Patients	Controls	Statistic Significance
Race (% Chinese)	100	140	-
Gender (% male)	44.29	55.71	0.848
Education (year)	10.60 ± 5.117	11.86 ± 5.416	0.071
Age	21.57 ± 12.850	21.08 ± 10.776	0.755
DMS-TC	18.31 (58)	15.593 (67)	0.049

DMS-TC: Delay-matching-to-sample total correct numbers.

### 2.2. Analysis of Quantitative Traits (QTs) and Over-Representation Study

**Figure 1:**
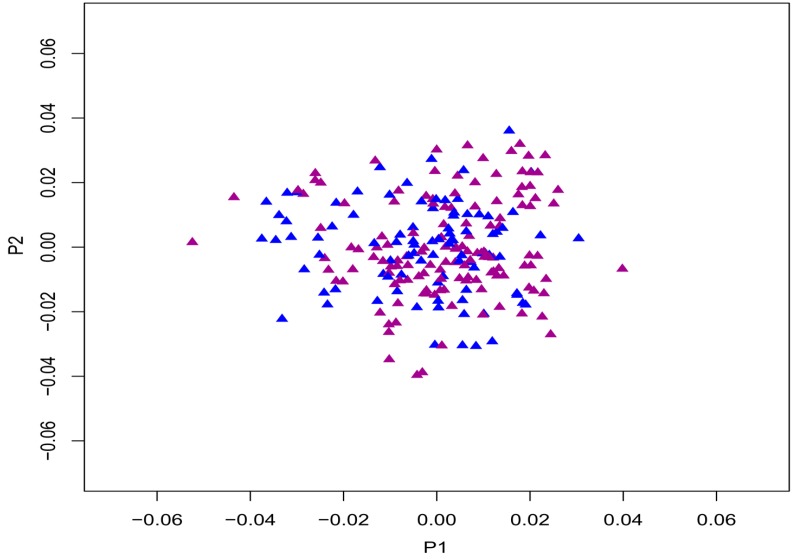
Multidimensional scaling plot of first two multidimensional scaling (MDS) components. Blue = control; Dark magenta = case.

**Figure 2:**
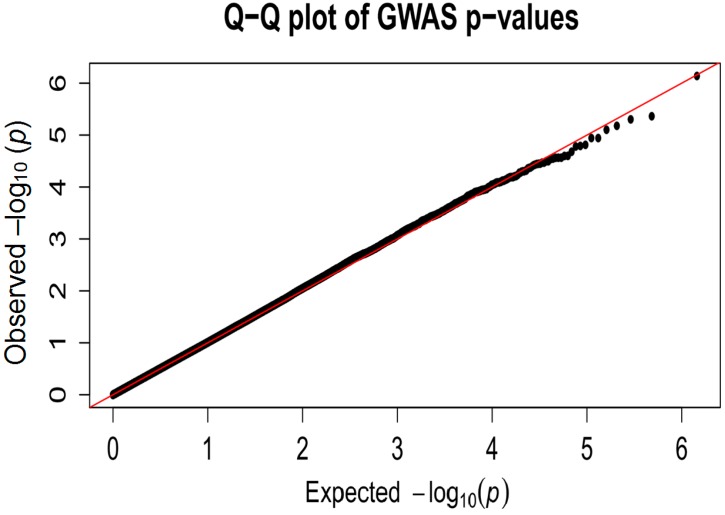
Q-Q plot of genome-wide association study (GWAS) on schizophrenia using DMS-TC (DMS-total correct numbers) as quantitative trait.

**Figure 3:**
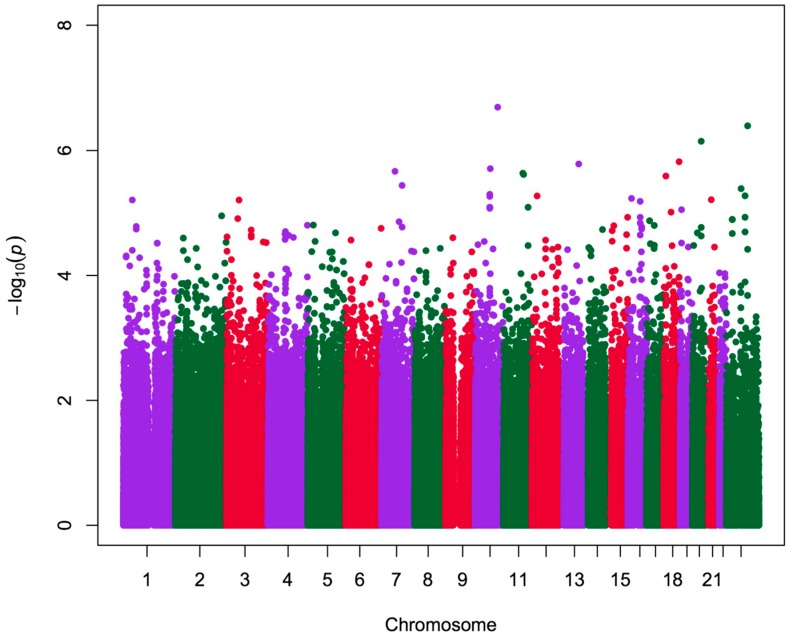
Manhattan plots of genome-wide association of all SNPs with DMS-TC. SNPs were plotted on the x axis according to their position on each chromosome represented by difference color bars against association of DMS-TC on the y axis (shown as −log10P value).

A total of 742,805 SNPs passed the quality control, with a mean call rate of 98.9%. However, seven patients and six controls failed to pass the quality control and cryptic relatedness, and thus were excluded from subsequent analysis. Multidimensional scaling (MDS) in PLINK showed that individuals were tightly clustered, indicating that individuals were of the same ancestral Chinese Han origin (Figure 1). Following quality control, inflation factor (λ), generated from PLINK analysis was found to be 1.0099, which showed that confounding factors were well-adjusted (Figure 2). Since there was no conspicuous population structure among the study samples and no significant deviation of the observed distribution, principal components from MDS was not chosen as a covariate for the linear regression analysis. Finally, genotyping data of 93 patients and 134 controls passed the quality control and were included for the subsequent association study. Results from the association test of genotype × group and DMS-TC QTs demonstrated that rs1411832 was found to be the most significant SNP, which was located at the downstream of YWHAZP5 (*p* = 2.02 × 10^−7^) (Table 2). 121 autosomal variants, located in or within flanking areas of 46 genes, passed the significant threshold of 5 × 10^−5^ using DMS-TC as quantitative trait (Figure 3). These annotated genes were used to map the significant pathways for the study. Pathway over-representation analysis of annotated genes by ConsensusPathDB showed that nine genes (SCARB1, DSCAM, LGALS4, COL14A1, PTPRT, IGSF11, DSCAML1, SMOC2, and FAT3) were significantly over-represented in one GO, cell adhesion (GO:0007155, *p* < 0.001).

**Table 2:** Significant interaction of SNPs × diagnosis and quantitative trait (DMS-TC) in DMS.

CHR	SNP	Position	Type	Gene	Traits	*p*
10	rs1411832	107886255	Intergenic	Downstream of YWHAZP5	DMS-TC	2.02 × 10^−7^
20	rs61131853	41749812	Intron	PTPRT	DMS-TC	7.10 × 10^−7^
18	rs79589976	73305576	Intron	TADA2L	DMS-TC	1.51 × 10^−6^
13	rs74108723	90358757	Intergenic	N/A	DMS-TC	1.64 × 10^−6^
10	rs10999524	72525761	intergenic	Upstream of C10orf27	DMS-TC	1.95 × 10^−6^
7	rs4718138	64303065	Intron	ZNF138	DMS-TC	2.15 × 10^−6^
11	rs1552511	92605986	Intron	FAT3	DMS-TC	2.31 × 10^−6^
11	rs555329	95993708	Intron	FAT3	DMS-TC	2.41 × 10^−6^
18	rs2868934	10204383	Intron	TADA2L	DMS-TC	2.56 × 10^−6^
7	rs60569161	98199903	intergenic	Upstream of NPTX2	DMS-TC	3.62 × 10^−6^
10	rs7899885	70143774	Intron	RUFY2	DMS-TC	5.02 × 10^−6^
12	rs12811916	25550866	Intron	DSCAML1	DMS-TC	5.33 × 10^−6^
10	rs2281698	70104320	Intron	RUFY2	DMS-TC	5.42 × 10^−6^
16	rs4780688	17567540	intergenic	Downstream of XYLT1	DMS-TC	5.84 × 10^−6^
21	rs76659985	26786931	Intron	LINC00158	DMS-TC	6.13 × 10^−6^
1	rs3738516	43440201	Intron	Downstream of SLC2A1	DMS-TC	6.19 × 10^−6^
3	rs17609699	60487578	Intron	FHIT	DMS-TC	6.20 × 10^−6^
16	rs4785000	58963145	intergenic	Upstream of LOC100132798	DMS-TC	6.51 × 10^−6^
11	rs630024	117534353	Intron	MAML2	DMS-TC	8.09 × 10^−6^
10	rs3781567	70105178	Intron	RUFY2	DMS-TC	8.11 × 10^−6^
10	rs1162753	70105560	synonymous	RUFY2	DMS-TC	8.11 × 10^−6^
10	rs17297439	70103461	Intron	RUFY2	DMS-TC	8.11 × 10^−6^
10	rs3199937	70102749	Intron	RUFY2	DMS-TC	8.11 × 10^−6^
10	rs3781568	70105286	Intron	HNRNPH3	DMS-TC	8.11 × 10^−6^
10	rs7897488	70179746	Intron	RUFY2	DMS-TC	8.11 × 10^−6^
10	rs7071140	70100250	Intron	HNRNPH3	DMS-TC	8.42 × 10^−6^
19	rs7249563	7009813	intergenic	Downstream of VAPA	DMS-TC	8.85 × 10^−6^
18	rs652630	34899378	Intron	CELF4	DMS-TC	9.70 × 10^−6^
2	rs520102	220529636	intergenic	Downstream of SLC4A3	DMS-TC	1.11 × 10^−5^
15	rs2453034	101241344	Intron	NPAS3	DMS-TC	1.17 × 10^−5^
16	rs6499996	58993725	intergenic	Downstream of XYLT1	DMS-TC	1.17 × 10^−5^
3	rs6773944	54516884	Intron	CACNA2D3	DMS-TC	1.23 × 10^−5^
17	rs2322973	13699188	intergenic	Downstream of LOC644649	DMS-TC	1.33 × 10^−5^
7	rs58908055	83788680	Intron	SEMA3A	DMS-TC	1.38 × 10^−5^
16	rs9930442	58978493	intergenic	Downstream of LOC644649	DMS-TC	1.45 × 10^−5^
5	rs59017736	26250801	intergenic	Downstream of MSNL1	DMS-TC	1.56 × 10^−5^
4	rs4470690	188721512	intergenic	Downstream of LOC644325	DMS-TC	1.57 × 10^−5^
17	rs8067120	35783565	Intron	TADA2A	DMS-TC	1.58 × 10^−5^
15	rs1458888	35406129	Intron	NPAS3	DMS-TC	1.60 × 10^−5^
1	rs1286830	62270494	Intron	INADL	DMS-TC	1.64 × 10^−5^
16	rs8060933	65891108	intergenic	Downstream of LOC644649	DMS-TC	1.67 × 10^−5^
7	rs705337	98226627	intergenic	Upstream of NPTX2	DMS-TC	1.68 × 10^−5^
20	rs6030661	41748131	Intron	PTPRT	DMS-TC	1.70 × 10^−5^
6	rs4708759	169005721	Intron	SMOC2	DMS-TC	1.76 × 10^−5^
16	rs12373039	65894066	intergenic	Downstream of LOC644649	DMS-TC	1.80 × 10^−5^
1	rs1286831	62271962	Intron	INADL	DMS-TC	1.81 × 10^−5^
14	rs78636353	88681552	Intron	KCNK10	DMS-TC	1.84 × 10^−5^
3	rs614673	118539108	intergenic	Upstream of IGSF11	DMS-TC	1.87 × 10^−5^
15	rs28436697	27624233	Intron	GABRG3	DMS-TC	1.92 × 10^−5^
4	rs77470375	83823703	Intron	THAP9	DMS-TC	1.98 × 10^−5^
5	rs2066960	131994435	Intron	IL13	DMS-TC	2.08 × 10^−5^
20	rs6132627	23444688	Intron	LGALS4	DMS-TC	2.08 × 10^−5^
4	rs62303604	82397503	intergenic	Downstream of RASGEF1B	DMS-TC	2.17 × 10^−5^
4	rs16998600	82403384	intergenic	Downstream of RASGEF1B	DMS-TC	2.17 × 10^−5^
4	rs62302363	82417252	intergenic	Downstream of RASGEF1B	DMS-TC	2.17 × 10^−5^
4	rs17005142	82402588	intergenic	Downstream of RASGEF1B	DMS-TC	2.17 × 10^−5^
4	rs17005144	82404274	intergenic	Downstream of RASGEF1B	DMS-TC	2.17 × 10^−5^
4	rs6819741	82403942	intergenic	Downstream of RASGEF1B	DMS-TC	2.17 × 10^−5^
15	rs2575426	96405795	intergenic	Downstream ofLOC441722	DMS-TC	2.22 × 10^−5^
16	rs12931857	58967112	intergenic	Upstream of CDH5	DMS-TC	2.25× 10^−5^
3	rs80028372	118805552	Intron	IGSF11	DMS-TC	2.26 × 10^−5^
4	rs7438406	99702148	intergenic	Downstream of BTF3L3	DMS-TC	2.28 × 10^−5^
20	rs2325606	41738666	Intron	PTPRT	DMS-TC	2.33 × 10^−5^
3	rs17659192	3108711	Intron	IL5RA	DMS-TC	2.41 × 10^−5^
3	rs12630657	118842442	Intron	IGSF11	DMS-TC	2.43 × 10^−5^
4	rs10034975	122175499	intergenic	Upstream of GPR103	DMS-TC	2.46 × 10^−5^
9	rs7041922	34938198	intergenic	Upstream of KIAA1045	DMS-TC	2.49 × 10^−5^
2	rs6544074	37634473	intergenic	Downstream of QPCT	DMS-TC	2.52 × 10^−5^
4	rs10015146	82401332	intergenic	Downstream of RASGEF1B	DMS-TC	2.65 × 10^−5^
6	rs2744229	25341580	Intron	LRRC16A	DMS-TC	2.71 × 10^−5^
12	rs12318900	66044284	intergenic	Upstream of KRAS	DMS-TC	2.72 × 10^−5^
15	rs9708085	27619217	Intron	GABRG3	DMS-TC	2.83 × 10^−5^
5	rs6894424	34732456	Intron	RAI14	DMS-TC	2.85 × 10^−5^
10	rs2503870	43796180	Intron	HNRNPH3	DMS-TC	2.86 × 10^−5^
3	rs6808187	175861931	intergenic	Upstream of LOC730168	DMS-TC	2.91 × 10^−5^
2	rs755300	241652703	Intron	KIF1A	DMS-TC	2.94 × 10^−5^
3	rs4687154	190304172	Intron	IL1RAP	DMS-TC	2.98 × 10^−5^
19	rs1353166	6992943	intergenic	BRUNOL4	DMS-TC	3.02 × 10^−5^
1	rs347272	162318498	Intron	NOS1AP	DMS-TC	3.04 × 10^−5^
1	rs11577628	162319524	Intron	NOS1AP	DMS-TC	3.04 × 10^−5^
1	rs347273	162317513	Intron	NOS1AP	DMS-TC	3.04 × 10^−5^
17	rs8065154	17614947	Intron	RAI1	DMS-TC	3.11 × 10^−5^
17	rs6502615	17612023	Intron	TADA2L	DMS-TC	3.11 × 10^−5^
10	rs12268934	13581758	intergenic	Downstream of RASGEF1A	DMS-TC	3.21 × 10^−5^
17	rs11263747	35742069	Intron	RAI1	DMS-TC	3.30 × 10^−5^
17	rs11263750	35816826	Intron	RAI1	DMS-TC	3.30 × 10^−5^
17	rs11868171	35816330	Intron	C17orf78	DMS-TC	3.30 × 10^−5^
17	rs2898656	35806418	Intron	ACACA	DMS-TC	3.30 × 10^−5^
20	rs2024886	5700696	Intron	PTPRT	DMS-TC	3.31 × 10^−5^
11	rs583983	117525125	Intron	DSCAML1	DMS-TC	3.32 × 10^−5^
18	rs72899323	39845104	Intron	LINC00907	DMS-TC	3.34 × 10^−5^
19	rs1035525	39299362	Intron	LGALS4	DMS-TC	3.50 × 10^−5^
12	rs10846743	125310305	Intron	SCARB1	DMS-TC	3.51 × 10^−5^
21	rs7283946	42143503	Intron	DSCAM	DMS-TC	3.51 × 10^−5^
14	rs10131813	23745533	Intron	HOMEZ	DMS-TC	3.58 × 10^−5^
14	rs10144278	23749595	Intron	HOMEZ	DMS-TC	3.58 × 10^−5^
15	rs72633609	96389640	intergenic	Upstream of LOC100132798	DMS-TC	3.66 × 10^−5^
15	rs11858405	96331346	Intron	GABRG3	DMS-TC	3.66 × 10^−5^
2	rs6544072	99112745	Intron	INPP4A	DMS-TC	3.68 × 10^−5^
8	rs10111291	121266654	Intron	COL14A1	DMS-TC	3.68 × 10^−5^
17	rs58509949	35016090	Intron	TADA2L	DMS-TC	3.70 × 10^−5^
12	rs7137152	66063249	Intron	SCARB1	DMS-TC	3.75 × 10^−5^
12	rs17120580	66018826	intergenic	Upstream of LOC204010	DMS-TC	3.75 × 10^−5^
10	rs2349764	85675348	intergenic	At upstream of PRPF18	DMS-TC	3.76 × 10^−5^
14	rs8005082	28802074	Intron	HOMEZ	DMS-TC	3.78 × 10^−5^
12	rs7968661	99137384	Intron	ANKS1B	DMS-TC	3.81 × 10^−5^
13	rs9315422	37055511	intergenic	Downstream of CCNA1	DMS-TC	3.86 × 10^−5^
1	rs4660239	43431528	Intron	SLC2A1-AS1	DMS-TC	3.95 × 10^−5^
14	rs1958005	33646022	Intron	HOMEZ	DMS-TC	3.97 × 10^−5^
2	rs6544072	37619430	intergenic	Downstream of QPCT	DMS-TC	4.01 × 10^−5^
8	rs7829966	53081817	Intron	ST18	DMS-TC	4.01 × 10^−5^
7	rs7794560	143029983	Intron	CLCN1	DMS-TC	4.06 × 10^−5^
3	rs17659353	3111436	Intron	IL5RA	DMS-TC	4.09 × 10^−5^
5	rs10059239	18879807	intergenic	Downstream of LOC646241	DMS-TC	4.15 × 10^−5^
7	rs3298	154685873	Intron	DPP6	DMS-TC	4.17 × 10^−5^
16	rs9924423	64526807	intergenic	Upstream of CDH5	DMS-TC	4.18 × 10^−5^
9	rs12237468	125491634	Intergenic	Downstream of OR1L4	DMS-TC	4.19 × 10^−5^
5	rs56196053	115759247	intergenic	Upstream of SEMA6A	DMS-TC	4.20 × 10^−5^
5	rs1480583	105022790	intergenic	Downstream of RAB9P1	DMS-TC	4.25 × 10^−5^
14	rs912857	33657980	Intron	NPAS3	DMS-TC	4.85 × 10^−5^
1	rs12024045	14297220	Intron	KAZN	DMS-TC	4.86 × 10^−5^

CHR: Chromosome; DMS-TC: Delay-matching-to-sample total correct numbers; SNP: Single nucleotide polymorphism.

## 3. Discussion

### 3.1. Association Study of Working Memory Deficit as QT

The current study is the first GWAS utilizing one of the composite scores of the working memory task paradigm as QT to map common variants associated with schizophrenia. In this study, significant differences in computerized DMS were found between schizophrenia patients and healthy controls. One Composite scores, DMS-TC, was therefore chosen as QTs to associate with interaction of genotypes × group. Additionally, genes containing variants of significant importance were mapped by one GO term using pathway database. Results of the study highlighted the validity of the method used for genetic underpinning in the pathology of schizophrenia.

We found rs1411832 with the smallest p value in one of the intergenic regions downstream of gene YWHAZP5 (tyrosine 3-monooxygenase/tryptophan 5-monooxygenase activation protein, zeta pseudogene 5). Most pseudogenes are very similar protein-coding genes and some are believed to be extra copies of genes; a recent study demonstrated that some pseudogenes are functional [18]. YWHAZ has been well-studied in schizophrenia [19,20,21], its gene product belongs to the 14-3-3 family of proteins which mediate signal transduction by binding to phosphoserine-containing proteins. 14-3-3 is abundant in brain which points to its critical role in neuronal functions [22,23]. Initial finding of association can be followed up by (i) sequence similarity between YWHAZ and YWHAZP5; (ii) better defining the nature of the potential association between YWHAZ and YWHAZP5; and (iii) attempting to replicate it in two additional samples.

Furthermore, some studies have shown that intergenic variants might play an important role in regulating expression of nearby genes [24,25] and they are also often in linkage disequilibrium with causal variants in the gene. Further study should be conducted on causal variants in LD with this intergenic variant. Meanwhile, Since the study was trait-associated GWAS and both environment and gene can contribute to complex traits, it is likely that this significant variant regulates the expression of YWHAZP5 through epigenetic progress. Thus further studies are required to validate this presumption.

### 3.2. Over-Representation of Genes in Single Pathway

Pathway over-representation analysis of annotated genes by ConsensusPathDB showed that nine genes (SCARB1, DSCAM, LGALS4, COL14A1, PTPRT, IGSF11, DSCAML1, SMOC2, and FAT3) were significantly over-represented in one GO, cell adhesion (GO:0007155, *p* < 0.001). Of these nine genes, DSCAM and DSCAML1 (Down’s syndrome cell adhesion molecule gene) are associated with Down’s syndrome. Various studies have demonstrated a relationship between DSCAM and neurobehavioral phenotype of Down’s syndrome which includes working memory deficit [26,27,28,29]. Recently, exome sequencing has detected genetic overlapping between schizophrenia and neurodevelopmental disorder [30,31]. From the above studies, we concluded that there should at least be a subgroup in schizophrenia that shares the same molecular pathological pathway with neurodevelopmental disorder. Beside this, PTPRT was often found to be related to neurodevelopment and cell growth [31,32]. A neurodevelopment model of schizophrenia has been frequently verified in studies involving different research strategies. Findings from our study add further evidence that neurodevelopmental deficit is associated with the pathogenesis of schizophrenia.

### 3.3. Functional Characterization of Genes Post GWAS

In recent years, various arguments were made on the extrapolation of the GWAS results in order to clarify the current vague picture. After GWAS, *a posteriori* functional pathway is one of the most authentic strategies to study the role of mapped genes in the complex disorder [33,34,35]. Pathway approaches have been adopted in many studies [36,37] and are shown to be robust to detect the joint action of variants of small effect clustering within biological pathways that play a major role in predisposing to complex genetic disorders and they can increase power by summarizing combined effects of all SNPs within a pathway in attempt to make biological meaningful interpretations of the data [38,39,40]. Even very large GWAS may lack power to identify small SNP effects, but these may be detectable at a pathway level.

In our study, subsequent functional enrichment from ConsensusPathDB highlighted that the tagged genes were mostly enriched in cell adhesion (GO:0007155). Cell adhesion molecules in nervous system and neural cell adhesion molecules (NCAMs) play a critical role in neural development such as cell adhesion, growth, and migration as scaffold for novel binding proteins [41,42,43], and various studies have demonstrated changes in NCAMs in schizophrenia patients. Although NCAM abnormalities in schizophrenia patients have been widely described, very few studies focus on the relationship between extracellular matrix and paradigm of the visual working memory test.

## 4. Experimental Section

### 4.1. Participants

One hundred first-episode schizophrenia patients (recruited from West China Hospital of Sichuan University, Chengdu, China) and 140 demographically matched healthy controls without family history of psychological disorders (recruited from the local neighborhood), were included in the study. Patients were interviewed with SCID (structured clinical interview) by trained psychiatrists to ensure the diagnosis was based on DSM-IV (Diagnostic and Statistical Manual of Mental Disorders, fourth edition) criterion, whereas healthy controls were screened with structured clinical interview-non-patient (SCID-NP) to ensure the absence of psychiatric illness. Patients were followed up to 6 months in order to confirm the diagnosis. No anti-psychotic medication was administered to patients at the time of clinical and neuro-cognitive evaluation. Both patients and controls were excluded if they had one of following conditions: (i) organic cerebral diseases; (ii) neurological diseases; (iii) severe endocrine diseases; (iv) axis I and II diagnosis other than schizophrenia according to DSM-IV; and (v) single or double limb palsy. Informed written consent was obtained from all the participants after explaining the study. This study was approved by the Institutional Review Broad (IRB) of West China Hospital, Sichuan University.

### 4.2. DMS Test Paradigm

DMS is one of the computerized tests in the Cambridge Neuropsychological Test Automated Battery (CANTAB) (available on line: http://www.cantab.com), which is used to assess working memory. The paradigm of the DMS test is described elsewhere in detail [44]. DMS test data was obtained for 125 out of 227 genotyped individuals (58 patients and 67 healthy controls). There are 19 scores from the DMS test which belongs to three categories: correctness in terms of number and percentage (total correct percentage, correct percentage on simultaneous level, correct percentage on all delays, correct percentage on 0 ms delay, correct percentage on 4000 ms delay, correct percentage on 12,000 ms delay, total correct numbers, total correct numbers on all delays, total correct numbers on simultaneous level, total correct numbers on 0 ms delay, total correct numbers on 4000 ms delay, total correct numbers on 12,000 ms delay); latency at both simultaneous and delayed level (mean correct latency, mean correct latency on all delays, mean correct latency on simultaneous level); and statistical analysis of discrimination between signal and noise (prob error given correct, prob error given error, DMS A’, DMS B’). DMS-TC, we are choosing here as QTs for association study, belongs to correctness in terms of number and percentage. It indicates the total number of trials in which subjects selected correct stimulus as their first response and is calculated by combination of total correct numbers on simultaneous level and total correct numbers on all delays.

### 4.3. Statistical Analysis

#### 4.3.1. DMS Test

Analysis of variance (ANOVA) using statistical package for the social sciences (SPSS21.0, SPSS, Inc., Chicago, IL, USA) was used to compare DMS test scores between patients and healthy controls, adjusting age, education years, and gender as covariates. The multiple test was accounted for by using Bonferroni correction. To justify the inter-group differences, statistically significant values (*p*-value) were set to *p* < 0.05 (Bonferroni correction).

#### 4.3.2. Genotyping and Quality Control

DNA was extracted from whole blood samples using the standard isolation method and the genotyping was performed on the HumanOmniZhongHua-8 Bead Chip platform. Genotyping data were systematically filtered according to their genotyping rates (per sample and per marker), minor allele frequency (MAF), and Hardy-Weinberg equilibrium tests (only in controls) in PLINK. Participants with low genotyping rate (<97%), markers with missing rate >5% per individual and/or with MAF >0.05, and markers that failed to pass Hardy-Weinberg equilibrium tests (*p* ≤ 0.00001) were excluded from the study. Furthermore, gender of each participant, with genotyping data on gender-specific loci, was confirmed by genotyping platform and the participants with the lower genotype call rate were excluded if pairs of participants with identical genotypes were found. After systematic filtering, 109,923 SNPs and four individuals (two patients and two healthy controls) were excluded based on the set threshold value.

#### 4.3.3. Correction for Population Stratification

For further analysis of the genetic relationship between SNPs and population structure within the sample, remaining SNPs were further pruned to ensure linkage equilibrium between the SNPs. SNPs with *r*^2^ > 0.5, were consequently excluded from the study (PLINK command—indep-pairwise 50 10 0.5). The multi-dimensional scaling algorithm (MDS) for cryptic relatedness in PLINK was applied to a pruned sample. Matrix generated from MDS was visualized by plotting R package (available on line: http://www.r-project.org). Thirty eight thousand five hundred and fifty seven SNPs and nine subjects (five cases and four healthy controls) were excluded after failing the genetic relationship test and cryptic relatedness. Inflation factor, lambda (λ) was estimated by calculating the mean of observed and expected chi-square test statistics to analyze the results of whole-genome association studies for over-dispersion due to population substructure and other confounding factors.

#### 4.3.4. Linear Regression Analysis

DMS-TC scores, which significantly differed between patients and health controls, were used as QTs in regression analysis. Of the total of 227 subjects with high-quality genotyping data, 125 with DMS-TC were included for QT analysis. The QT analysis was based on comparing the differential effects of SNPs on the DMS QTs. Out of the four possible models (additive, co-dominant, dominant, and recessive), additive components (reflecting the additive contribution of risks for complex diseases) were included in the linear model. Multivariate linear regression model in PLINK1.07 [45] was used to assess the correlation of QT and interaction of group and additive genetic risk of minor allele of 742,805 SNPs which passed genetic quality control while adjusting for age, sex, and education year. The group was labeled as discrete variable (1 = patients, 2 = healthy controls) to define the interaction with the genetic risk of allele. Finally, a Manhattan plot was plotted using R to illustrate the whole genome-wide association scans. 

### 4.4. Over-Representation Study

To map the pathway over-represented by genes from association study, genes with SNPs passing a threshold of 5 × 10^−5^ were further explored by web server of ConsensusPathDB (available on line: http://cpdb.molgen.mpg.de/CPDB) to detect the set most enriched by these genes. Hyper-geometric test detect the gene set in predefined lists of functionally associated genes (pathways, gene ontology categories, and neighborhood-based entity sets) with *p*-value indicating the extent of corresponding enrichment. Previous studies shown that the *p* value of 5 × 10^−8^ is rather conservative which may lead to excessive false negative especially for gene- or pathway- based analysis in smaller sample size [46]. Some genes missed in the single-locus association test can be detected significantly when integrated into pathway association analysis [47,48,49]. In addition, Nicolae *et al.* [50] found that trait-associated SNPs below 5 × 10^−4^ are more likely to be eQTLs, and these signals from GWA study are not exhausted after fully investigating the results of expression quantitative trait locus (eQTL) studies in lymphoblastoid cell lines from HapMap samples. Enlightened by these studies, we set 5 × 10^−5^ as the empirical cut-off *p* value to identify potential pathways involved in working memory deficit in schizophrenia.

## 5. Conclusions

In our study, GWAS was conducted to find the genes that were significantly associated with working memory deficit of schizophrenia. The results showed that variants near one gene, which encodes YWHAZP5, were most significantly associated with working memory deficits in schizophrenia patients. Additionally, downstream pathway analysis showed that NCAM is essential in working memory-related pathogenesis of schizophrenia.

Although the study was promising in various aspects, there are some limitations associated with it. First, we chose only one part of results from DMS, *i.e.*, DMS-TC, as our QTs. We are fully aware that there are scores of many kinds generated from DMS of CANTAB, we multiple-corrected all *p*-values generated from DMS test using Bonferroni correction, and only the total correct numbers of the DMS could survive after the correction, which imply the strongest effect size in all indicators of the DMS. The other indicators could not survive after Bonferroni correction, so they were not included in subsequent analysis.

Second, this study is an explorative study of methodology involving variant mapping and annotation of its related biological pathway. SNP with the most significant *p*-value in our study is rs1411832 in chromosome 10 (*p* < 2.02 × 10^−7^), which is acceptable considering our sample size and missing phenotype data [51]. However, given the limitations of GWAS and its sample size, larger sample size with inclusion of both common and rare variants is required to validate the results of this study. Additionally, patients included in the study were all first-episode and drug-naïve. Some of them even reported difficulty in completing the task, thus resulting in missing phenotype data; given the study design, this is an inevitable trade-off for not being cofounded by medication and chronicity of the disease. In summary, this study showed that working memory might be one of the endophenotypes of schizophrenia and these endophenotypes can increase the efficiency of GWAS in neuropsychiatric disorders such as schizophrenia.
